# Supplementary material for: Structural basis of bile salt extrusion and small-molecule inhibition in human BSEP
Source: Nat Commun. 2023 Nov 10;14:7296. doi: 10.1038/s41467-023-43109-1 (PMC10638440; doi:10.1038/s41467-023-43109-1)
Supplement: Supplementary file 1 — Supplementary Information [file 41467_2023_43109_MOESM1_ESM.pdf]

Supplementary Information

Structural basis of bile salt extrusion and small-molecule inhibition in human BSEP

Hongtao Liu[1,3], Rossitza N. Irobalieva[1,3], Julia Kowal[1], Dongchun Ni[2], Kamil Nosol[1], Rose Bang-Sørensen[1], Loïck Lancien[1], Henning Stahlberg[2], Bruno Stieger[1] & Kaspar P. Locher[1,*]

[1]Institute of Molecular Biology and Biophysics, ETH Zürich, Zürich, Switzerland

[2]Laboratory of Biological Electron Microscopy, Institute of Physics, School of Basic Science, EPFL, and Department of Fundamental Microbiology, Faculty of Biology and Medicine, University of Lausanne, Lausanne, Switzerland

[3]These authors contributed equally: Hongtao Liu, Rossitza N. Irobalieva

*To whom correspondence may be addressed. email: locher@mol.biol.ethz.ch

This PDF file includes:
Supplementary Figures 1-14
Supplementary Tables 1-3
Supplementary References

Supplementary Figures

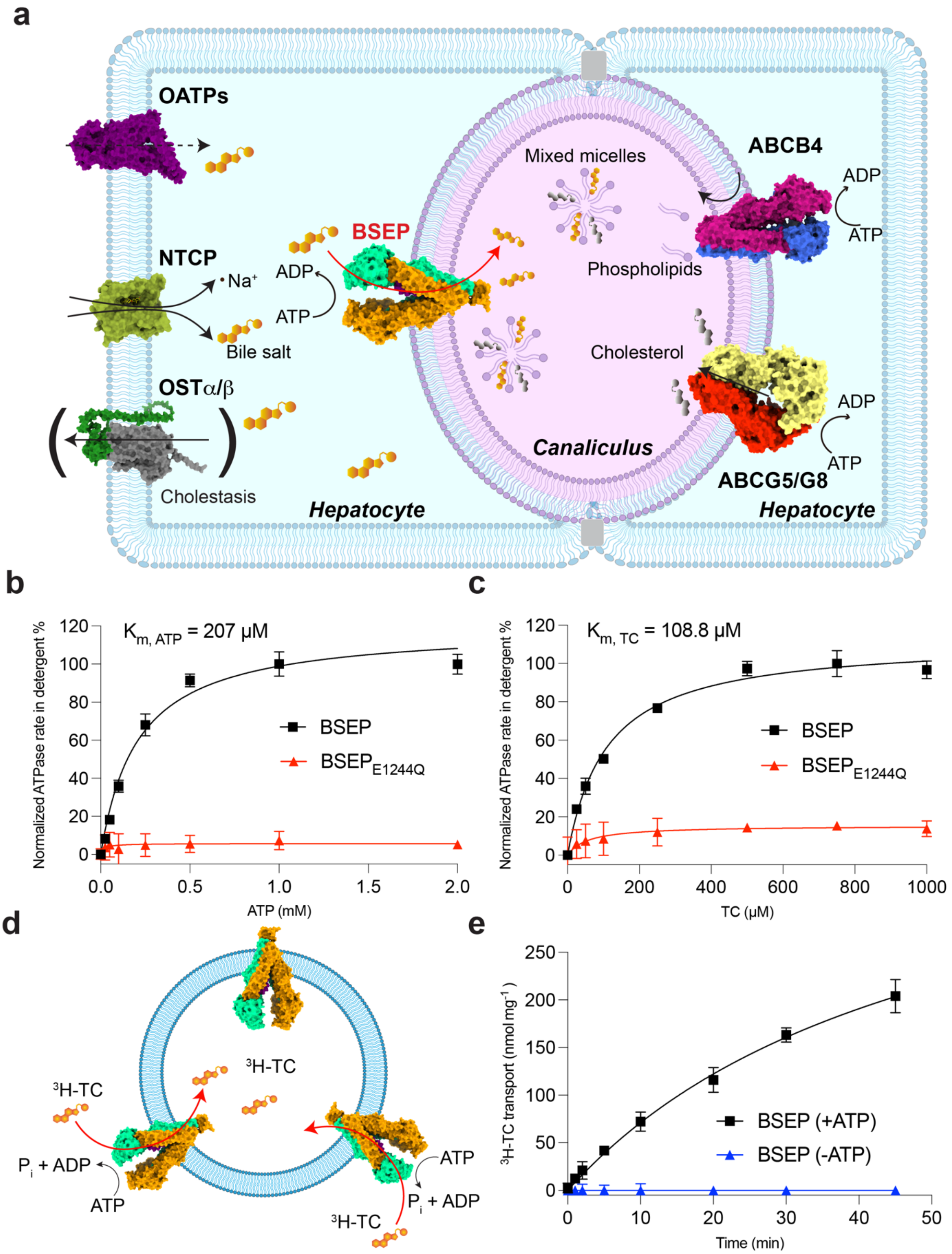

Supplementary Fig. 1. Functional activity of BSEP. a Overview of hepatocellular bile salt transport. Bile salts uptake into hepatocytes is mediated by Na^+ taurocholate co-transporting polypeptide (NTCP) and organic anion transporting polypeptides (OATPs) located in the

basolateral membrane, while BSEP mediates efflux across the canalicular membrane. The transporter OSTα/β has a low expression level in normal hepatocytes but can be up-regulated during cholestasis[1,2]. Surface representation of NTCP (PDB ID: 7ZYI), BSEP (PDB ID: 6LR0), ABCB4 (PDB ID: 7NIU), and ABCG5/8 (PDB ID: 7JR7) are indicated. The structures of OATPs (OATP1B3) and OSTα/β were downloaded from the AlphaFold Protein Structure Database (alphafold.ebi.ac.uk). **b, c** Normalized ATPase activity of BSEP and $BSEP_{E1244Q}$ in detergent as a function of ATP and TC concentration, respectively. **d** Schematic of the ^{3}H-TC transport assay in proteoliposomes. **e** Transport kinetics of BSEP mediated ^{3}H-TC transport in the presence (black curve) and absence (blue curve) of 5 mM ATP. All the data points indicate the mean of three independent measurements and error bars indicate SD.

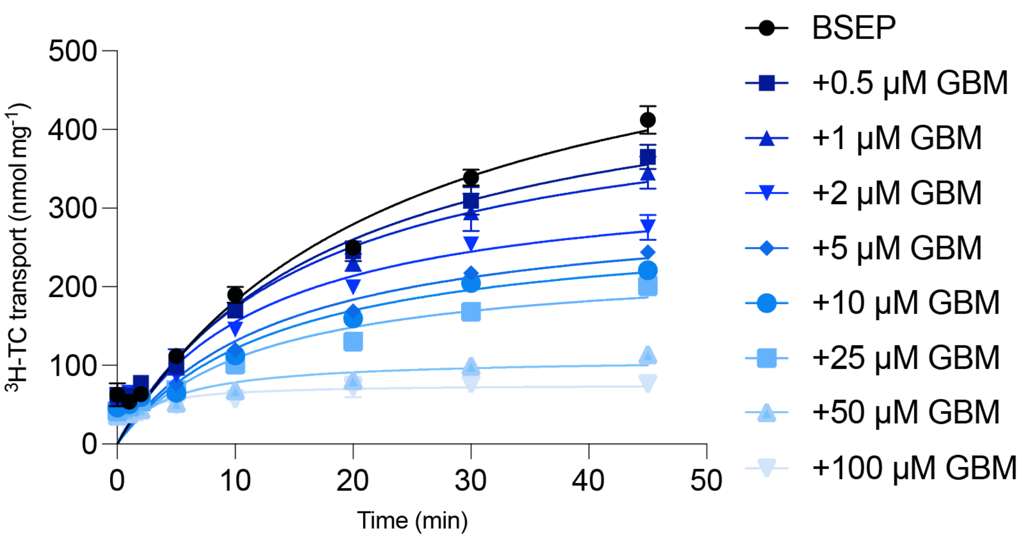

Supplementary Fig. 2. Functional characterization of BSEP with GBM. Transport kinetics of BSEP mediated ^{3}H-TC uptake with increasing GBM concentration in the presence of 50.5 □M TC. 5 mM ATP and 10 mM Mg^{2+} were added to initiate the reaction. All the data points indicate the mean of three independent measurements and error bars indicate SD.

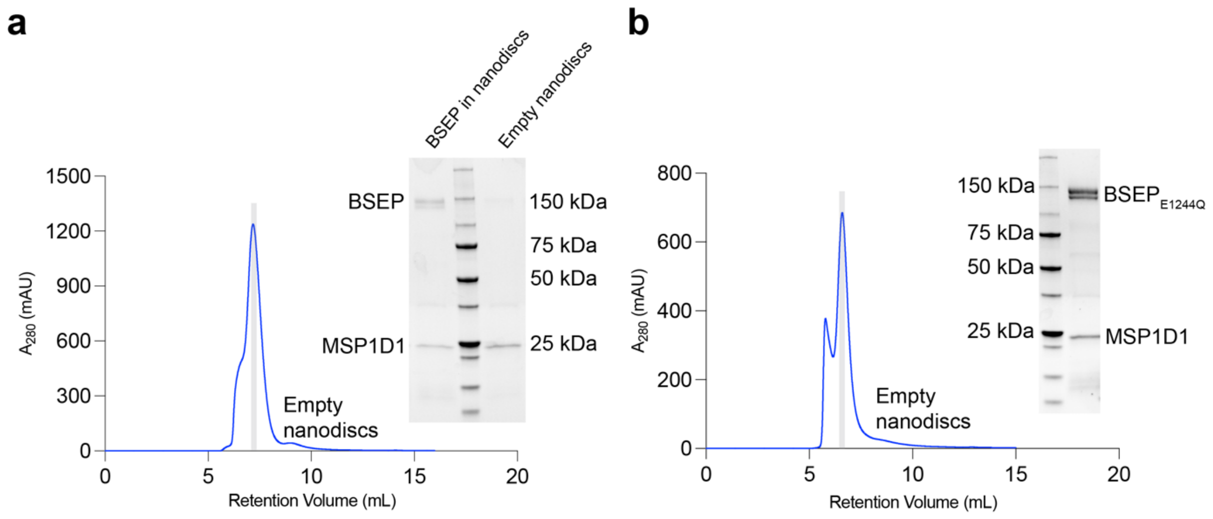

Supplementary Fig. 3. BSEP purification and nanodiscs reconstitution. Size-exclusion chromatography (SEC) profile of (**a**) wild-type BSEP and (**b**) $BSEP_{E1244Q}$ reconstituted in nanodiscs. A_{280}, absorbance at 280 nm. The grey-labeled fractions were used for cryo-EM sample preparation. Inset: The purified sample after SEC loaded on SDS-PAGE for BSEP or $BSEP_{E1244Q}$ and the membrane scaffold protein (MSP1D1).

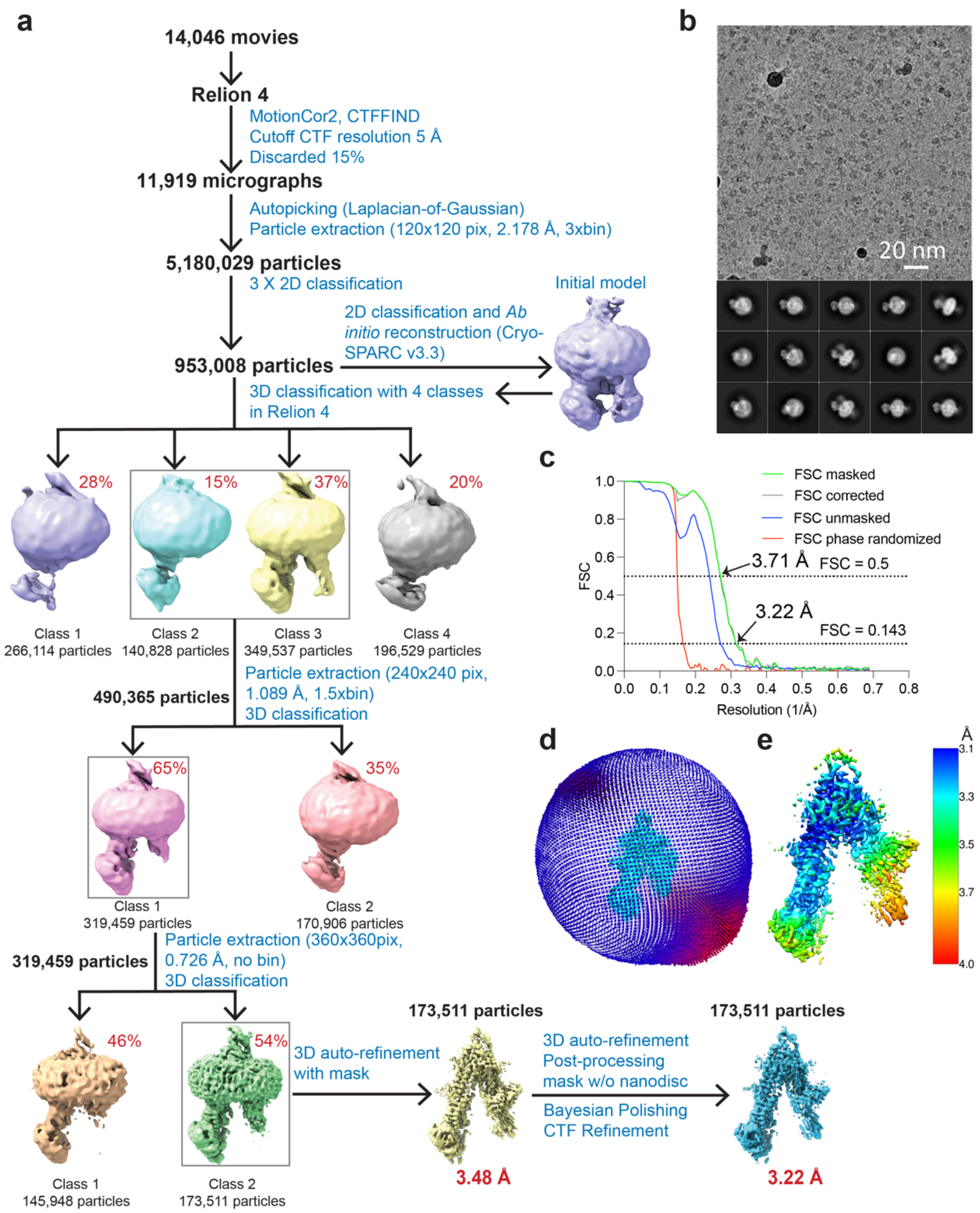

Supplementary Fig. 4. Cryo-EM data processing of GBM-bound BSEP. a Data processing flowchart of the GBM-bound structure of BSEP performed in CryoSPARC v3.3 and Relion 4.0. **b** Representative motion-corrected micrograph and 2D class averages of GBM-bound BSEP. **c** Fourier shell correlation (FSC) curves from Relion 4.0. **d** Angular distribution plot for the final reconstruction. **e** Local resolution estimation of the final EM density map generated in Relion 4.0.

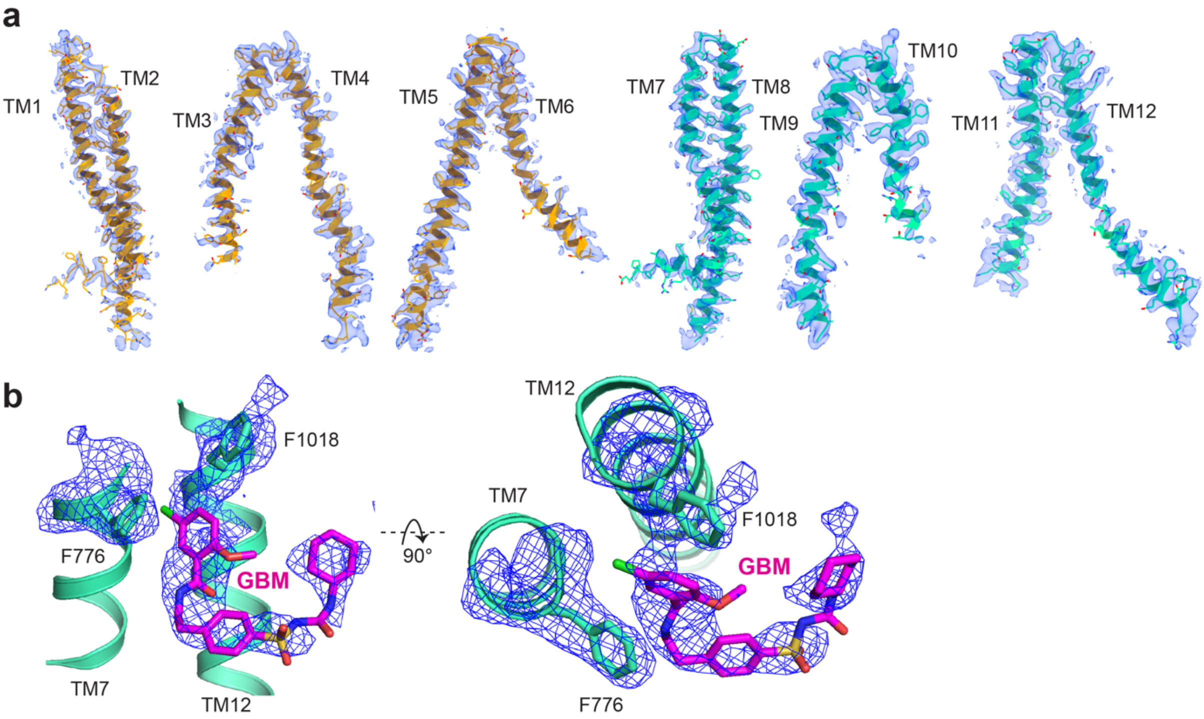

Supplementary Fig. 5. Cryo-EM density of GBM-bound BSEP. a BSEP TM helices and **b** GBM molecule (cartoon) and surrounding density are shown. The map is shown in the blue mesh with a contour level of up to 2 Å around the inhibitor. TM helices and residue numbers are indicated.

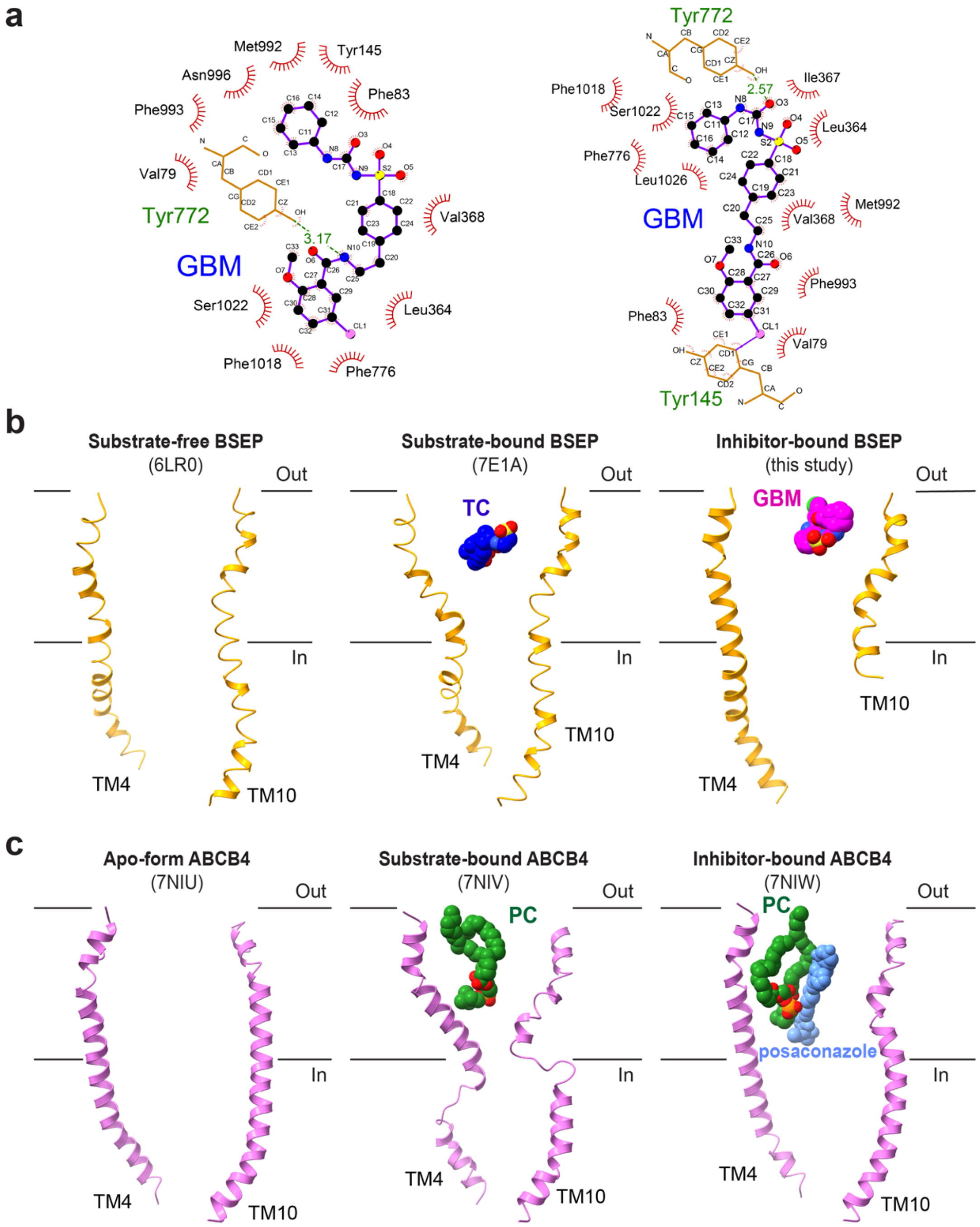

Supplementary Fig. 6. Binding of GBM in the pocket of BSEP. a Schematic diagram of the interactions between BSEP and GBM in two orientations. **b, c** Close-up views of TM4 and TM10 in BSEP and ABCB4. Bound TC and GBM (BSEP) are shown as spheres and colored blue and magenta, respectively. Bound PC and posaconazole (ABCB4) are shown as spheres and colored dark green and cornflower blue, respectively.

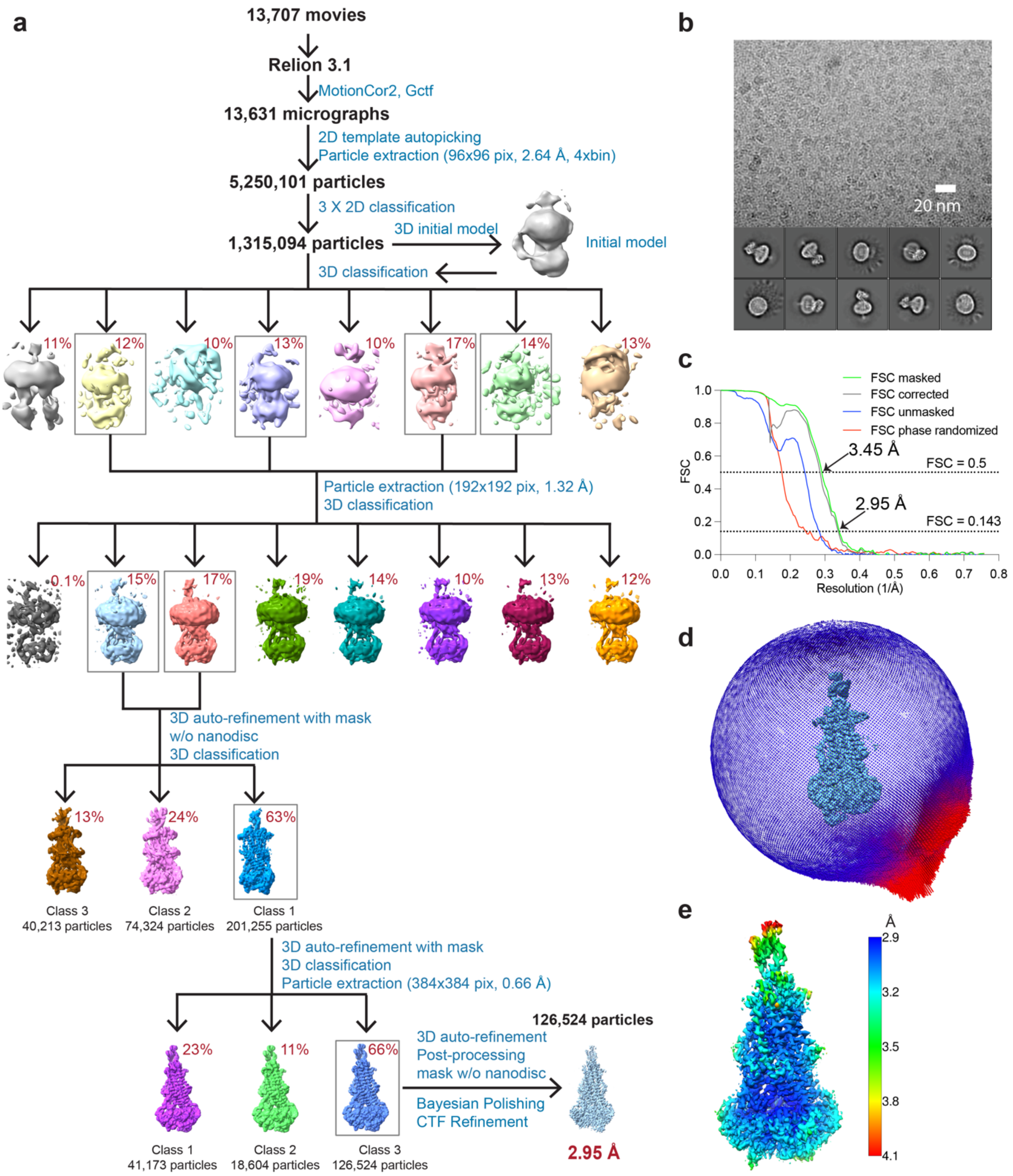

Supplementary Fig. 7. Cryo-EM data processing of ATP-bound BSEP$_{E1244Q}$. a Data processing flowchart of BSEP$_{E1244Q}$ performed in Relion 3.1. **b** Representative motion-corrected micrograph and 2D classes of BSEP$_{E1244Q}$. **c** Fourier shell correlation (FSC) curves from Relion 3.1. **d** Angular distribution plot for the final reconstruction. **e** Local resolution estimation for the final EM density map generated in Relion 3.1.

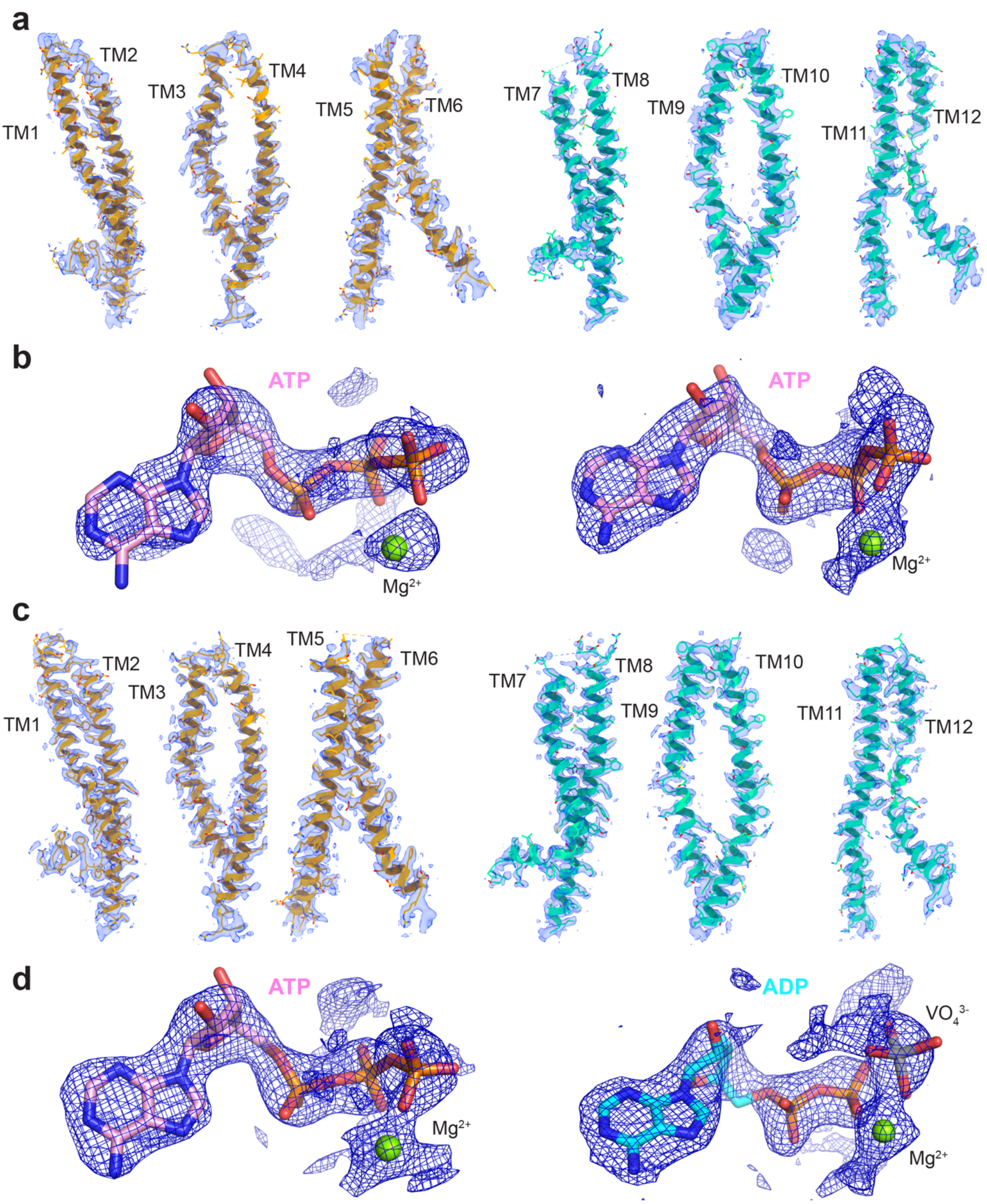

Supplementary Fig. 8. Cryo-EM density of BSEP$_{E1244Q}$ and vanadate-trapped BSEP. a, b Densities are shown for all six helices pairs of BSEP$_{E1244Q}$, as well as the two ATP (pink) molecules. **c, d** Densities are shown for all six helices pairs of vanadate-trapped BSEP, as well as for ATP (pink) and ADP (cyan) molecules.

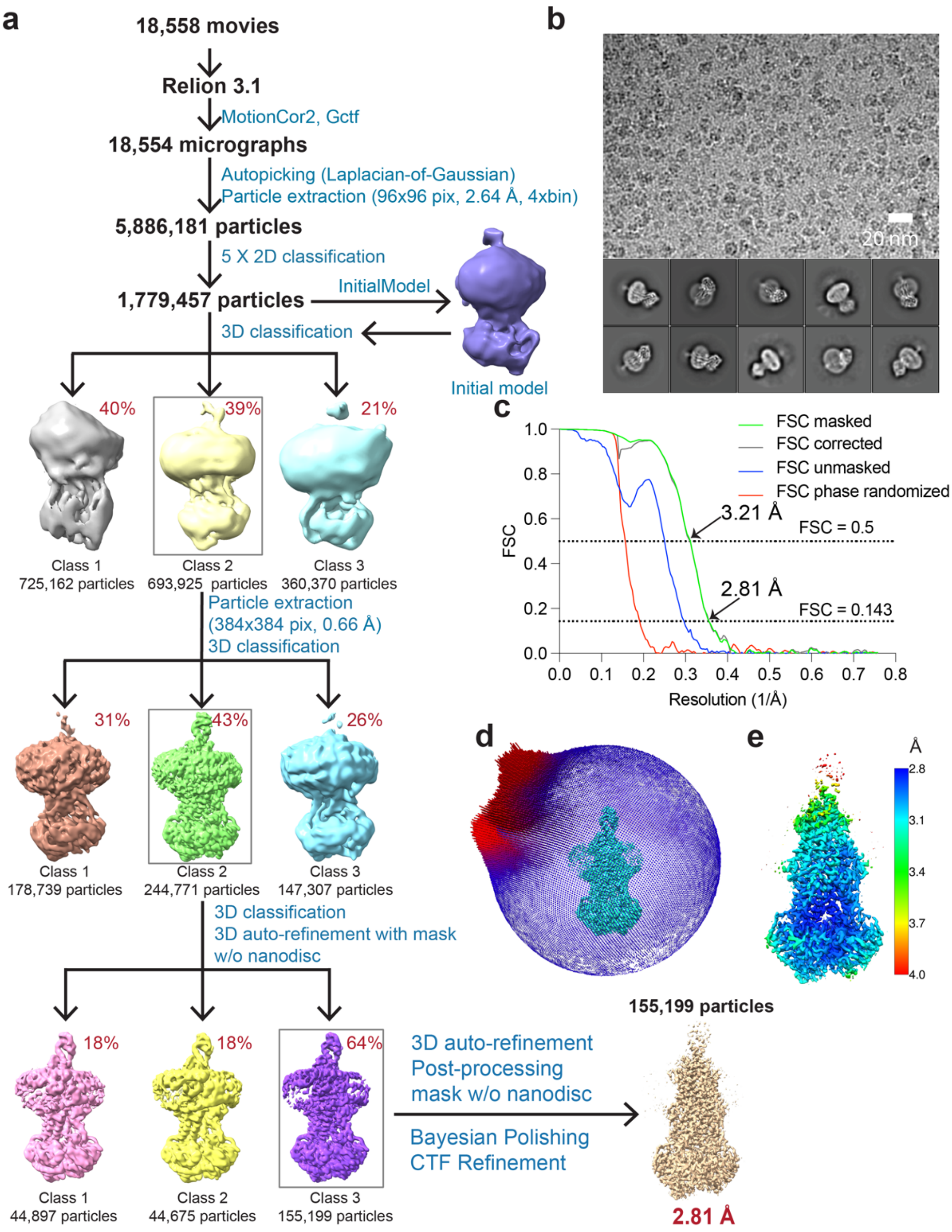

Supplementary Fig. 9. Cryo-EM data processing of vanadate-trapped BSEP. a Data processing flowchart of vanadate-trapped BSEP performed in Relion 3.1. **b** Representative motion-corrected micrograph and 2D class averages of vanadate-trapped BSEP. **c** Fourier shell correlation (FSC) curves from Relion 3.1. **d** Angular distribution plot for the final reconstruction. **e** Local resolution estimation for the final EM density map generated in Relion 3.1.

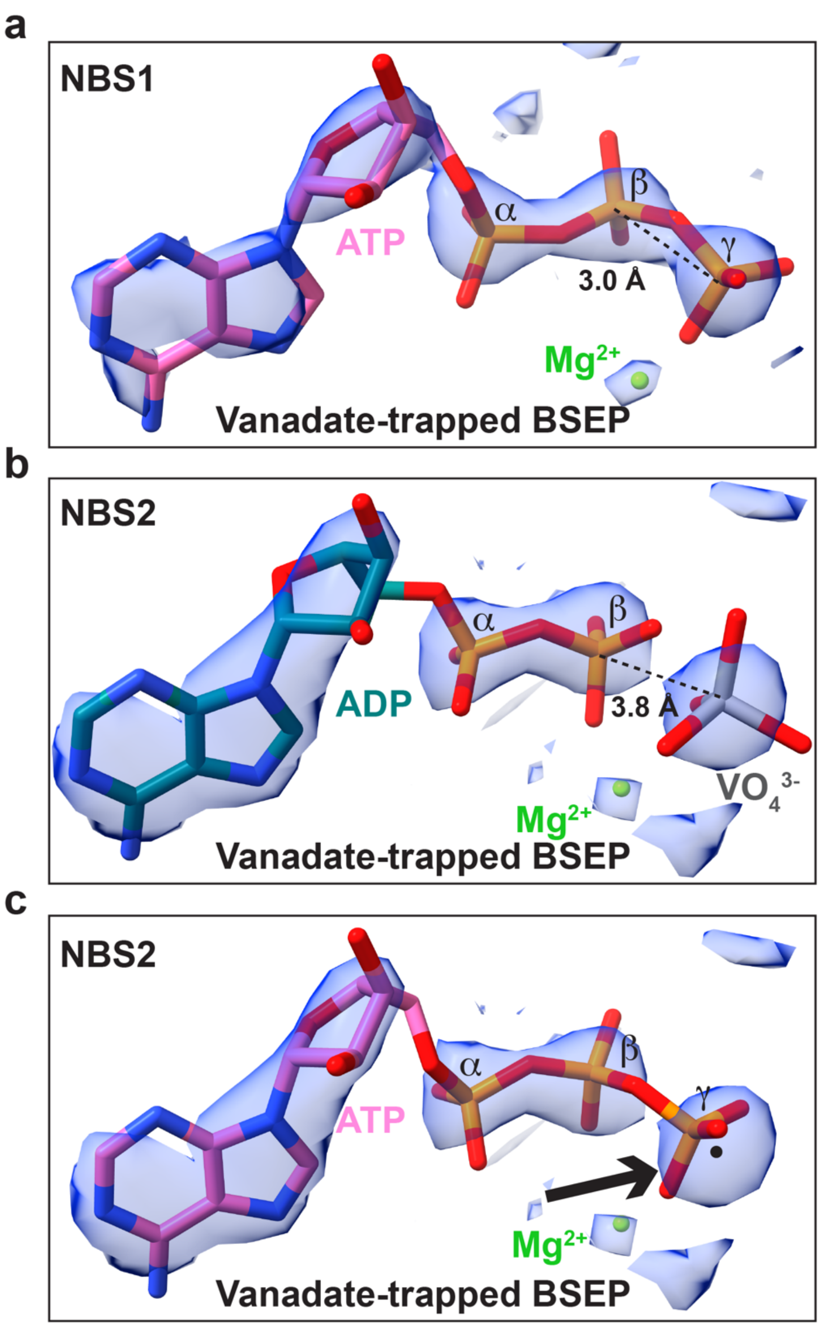

Supplementary Fig. 10. Nucleotides modeled in the EM map of vanadate-trapped BSEP. **a**, **b** Cryo-EM densities of nucleotides in both NBSs. ATP molecule fits in NBS1 and ADP-vanadate molecules in NBS2. The distances are measured and indicated in **a** and **b**. **c** Putative ATP molecule in the NBS2. The black dot was used to mark the center of the EM density corresponding to the vanadate. The ATP molecule is shorter than ADP-vanadate (shown in **b**) and does not fit well into the EM density (shown by the black arrow in **c**). All EM densities in **a**-**c** are shown at the same contour level.

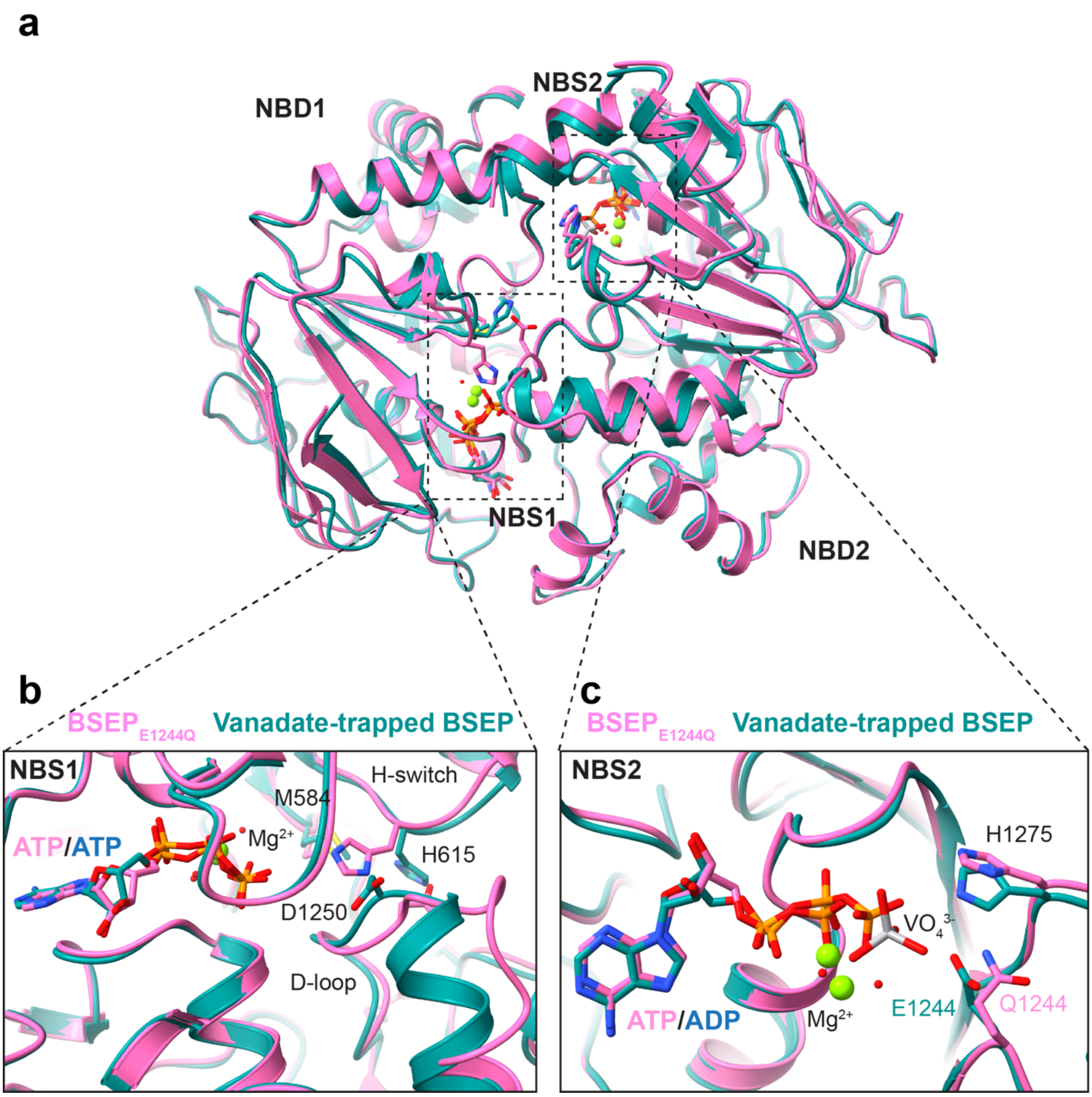

Supplementary Fig. 11. Close-up of the ATPase sites of $BSEP_{E1244Q}$ and vanadate-trapped BSEP. a Cartoon representation of the NBDs of $BSEP_{E1244Q}$ and vanadate-trapped BSEP viewed from the membrane toward the cytoplasm. NBS1 and NBS2 are indicated by the dashed line boxes. ATP and ADP are represented in sticks, and Mg^{2+} as green spheres. **b, c** The molecular interactions at NBS1 and NBS2 of $BSEP_{E1244Q}$ and vanadate-trapped BSEP.

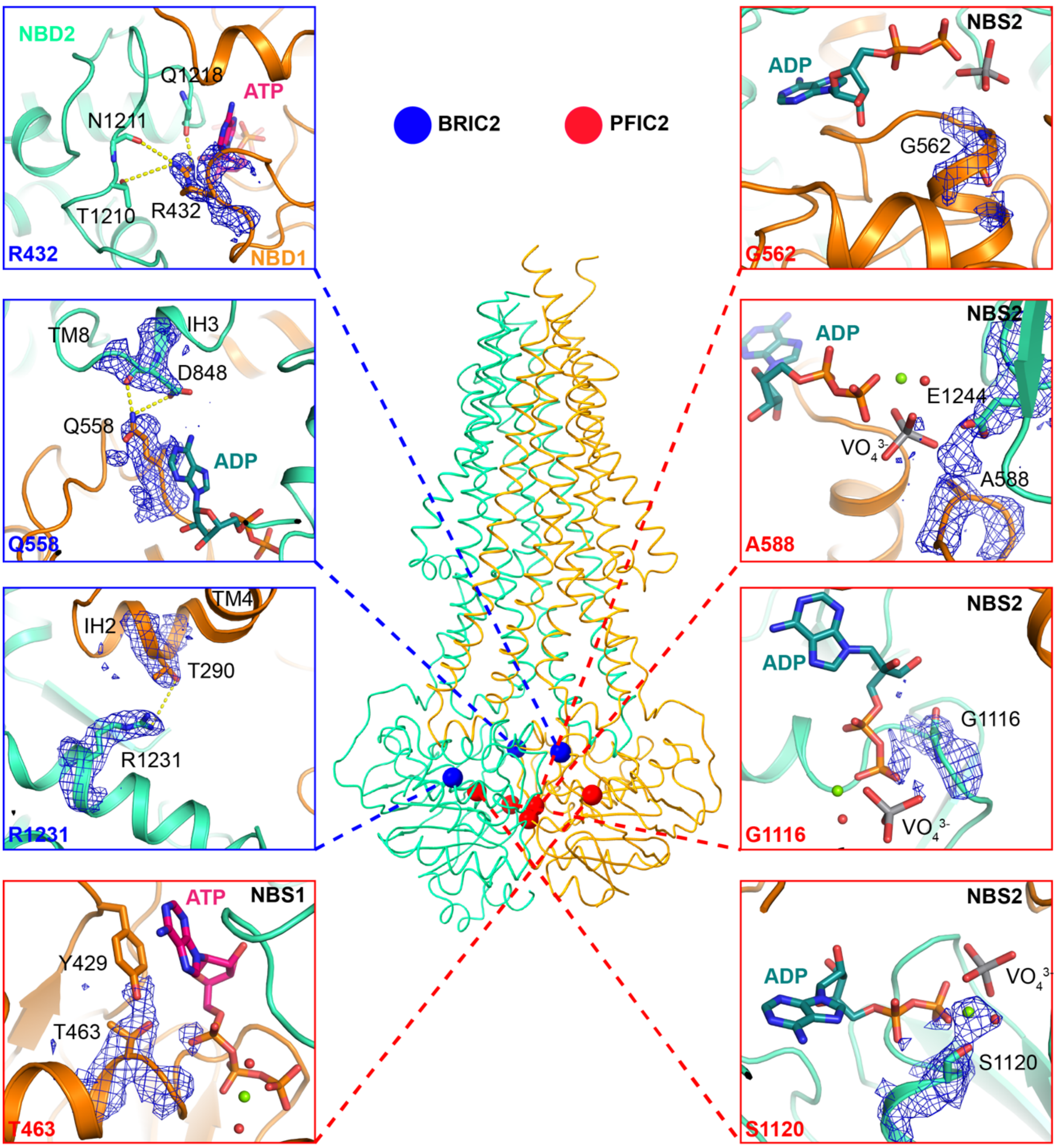

Supplementary Fig. 12. Disease-causing mutations in the NBDs. Localization of eight disease-causing mutations involved in PFIC2 (red sphere) and BRIC2 (blue sphere) in the NBDs. BSEP in collapsed conformation is shown as a ribbon. The molecular interactions of disease-causing mutations in NBDs are indicated, together with the density (blue mesh).

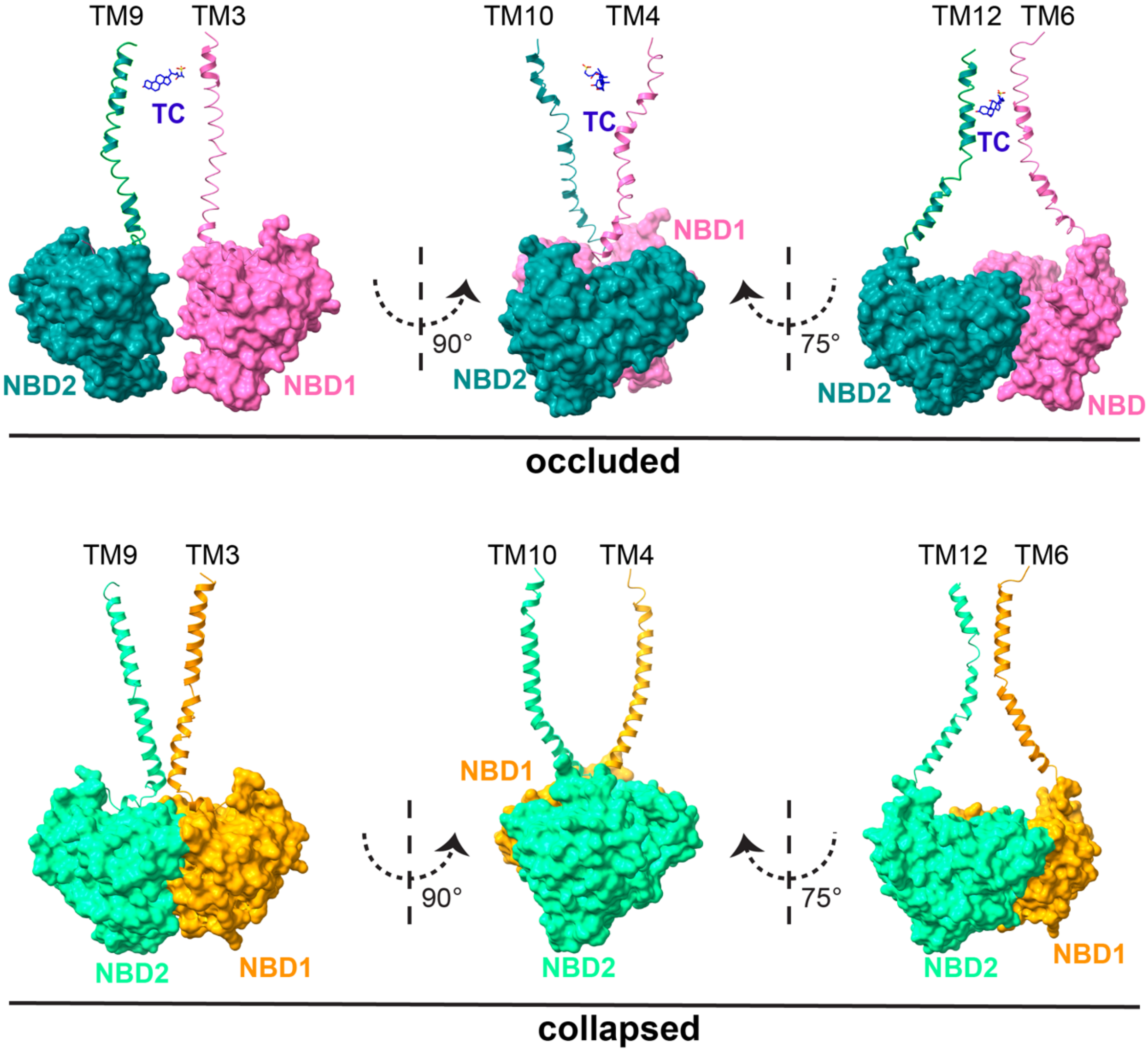

Supplementary Fig. 13. Movements of TM helices during substrate extrusion in BSEP. (Top panel) The role of TM helices in stabilizing substrate in TC-bound state (PDB: 7E1A). (Bottom panel) The role of TM helices in ATP-bound state ($BSEP_{E1244Q}$). TM helices are shown as cartoons and the NBDs are shown as surface.

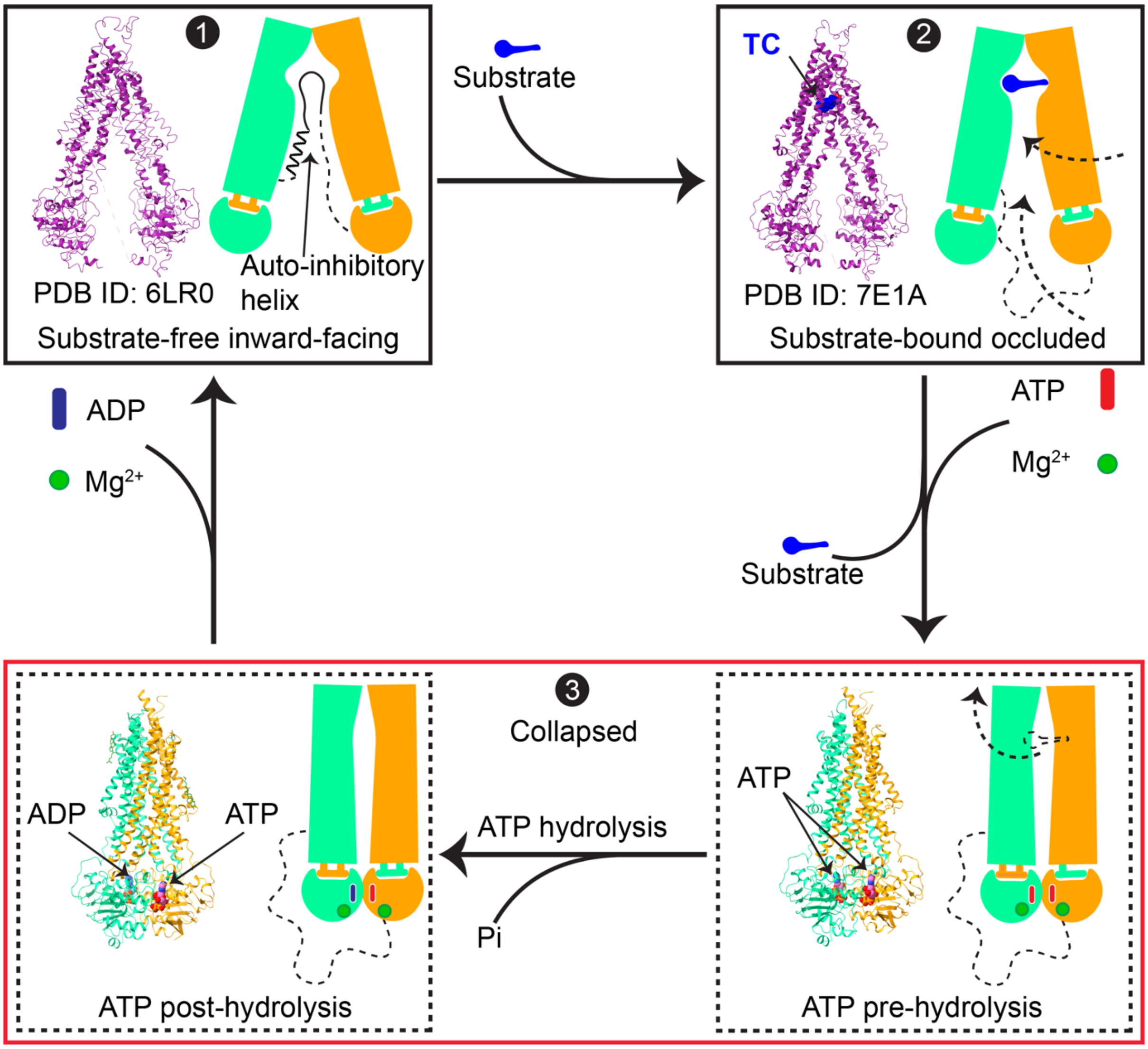

Supplementary Fig. 14. Bile salt transport cycle through BSEP. Schematic of the proposed bile salt transport cycle. The N- and C-terminal halves of BSEP are colored orange and green, respectively. The numbers in the black circles represent the functionally relevant conformational states of BSEP: state 1 and state 2 are based on PDB IDs: 6LR0 and 7E1A; state 3 represents the structures from this study. The substrate, inhibitor, ATP, ADP, Mg^{2+}, and VO_4^{3-} are shown.

Supplementary Table 1. Cryo-EM data collection and processing.

**Sample**	$BSEP_{E1244Q}$	Vanadate-trapped BSEP	GBM-bound BSEP
PDB	8PMD	8PMJ	8PM6
EMDB	EMD-17759	EMD-17761	EMD-17758
**Data collection and processing**			
Microscope,<br>Electron gun,<br>Energy filter,<br>Detector,	Titan Krios G3i,<br>X-FEG,<br>GIF BioQuantum<br>Gatan K3	Titan Krios G3i,<br>X-FEG,<br>GIF BioQuantum<br>Gatan K3	Titan Krios G4,<br>Cold FEG,<br>SelectrisX,<br>Falcon 4
Voltage (kV)	300	300	300
Magnification	130,000x	130,000x	165,000x
Electron exposure per movie ($e^-/Å^2$)	48	66	60
Defocus range (□m)	-0.6 to -2.4	-0.6 to -2.4	-0.8 to -2.5
Pixel size (Å)	0.66	0.66	0.726
Numbers of micrographs	13,631	18,554	14,046
Symmetry imposed	C1	C1	C1
Initial particle images (no.)	5,250,101	5,886,181	5,180,029
Final particle images (no.)	126,524	155,199	173, 511
Map resolution (Å) at FSC threshold of 0.143	2.95	2.81	3.22
Map sharpening *B* factor ($Å^2$)	-73.0	-46.6	-50.0
Processing software	Relion 3.1	Relion 3.1	Relion 4.0 and CryoSPARC v4.1
**Refinement**			
Initial model used (PDB code)	None	None	6LR0
FSC threshold	0.5	0.5	0.5
Model composition			
Non-hydrogen atoms	8,844	8,985	7,029
Protein residues	1,129	1,133	872
Ligands	Mg^{2+}: 2<br>ATP: 2	VO_4^{3-}: 1 ATP: 1<br>Mg^{2+}: 2 CLR: 4<br>ADP: 1	GBM: 1<br>CLR: 8
B factor ($Å^2$)			
Protein	2.75/89.81/35.41	4.13/111.46/35.47	41.90/268.58/122.42
Ligand	1.89/38.18/27.30	22.29/66696.90/11401.92	33.85/132.59/101.41
R. m. s. d deviations			
Bond lengths (Å)	0.004	0.003	0.005
Bond angles (°)	0.658	0.632	0.789
**Validation**			
MolProbity score	1.63	1.47	1.99
Clash score	13.15	6.92	14.16
Poor rotamers (%)	0.21	0	0.42
Ramachandran plot			
Favored (%)	98.21	97.60	95.24
Allowed (%)	1.79	2.40	4.76
Disallowed (%)	0.00	0.00	0.00

Supplementary Table 2. Structural alignment of $BSEP_{E1244Q}$, vanadate-trapped BSEP, $ABCB1_{EQ}$, and $ABCB4_{EQ}$.

Alignment	RMSD values (Å)			
	$BSEP_{E1244Q}$	Vanadate-trapped BSEP	$ABCB1_{EQ}$	$ABCB4_{EQ}$
$BSEP_{E1244Q}$		0.426	1.328	1.018
Vanadate-trapped BSEP			1.496	1.134
$ABCB1_{EQ}$				1.012
$ABCB4_{EQ}$				

Supplementary Table 3. Synopsis of eight disease-causing mutants of BSEP.

Mutants	Location	Disease	Defect	Reference
R432T	NBD1 and NBD2 interface	BRIC2	Reduced or absent bile salt transport	[3,4]
T463I	NBS1	PFIC2	Impaired ATP-binding, BSEP dysfunction	[3,5]
Q558H	NBS2 and IH3 interface	BRIC2	Reduced bile salt transport	[3,6]
G562D	NBS2	PFIC2	Impaired ATP-binding, BSEP dysfunction	[7]
A588V	NBS2	PFIC2	Impaired ATP-binding and hydrolysis	[7,8]
G1116R	NBS2	PFIC2	Impaired ATP-binding, BSEP dysfunction	[3,7]
S1120N	NBS2	PFIC2	Impaired ATP-binding and hydrolysis	[7]
R1231Q	NBD2 and IH2 interface	BRIC2	Reduced bile salt transport	[7,9]

Supplementary Reference

1. Boyer, J. L. *et al.* Upregulation of a basolateral FXR-dependent bile acid efflux transporter OSTalpha-OSTbeta in cholestasis in humans and rodents. *Am J Physiol Gastrointest Liver Physiol* **290**, G1124-1130 (2006).

2. Ueno, Y., Moritoki, Y., Shimosegawa, T. & Gershwin, M. E. Primary biliary cirrhosis: what we know and what we want to know about human PBC and spontaneous PBC mouse models. *J Gastroenterol* **42**, 189-195 (2007).

3. Kubitz, R., Dröge, C., Kluge, S., Stindt, J. & Häussinger, D. Genetic variations of bile salt transporters. *Drug Discov Today Technol* **12**, e55-67 (2014).

4. Noe, J. Kullak-Ublick, G. A. Jochum, W. Stieger, B. Kerb, R. Haberl, M. Müllhaupt, B. Meier, P. J. Pauli-Magnus, C. Impaired expression and function of the bile salt export pump due to three novel ABCB11 mutations in intrahepatic cholestasis. *J Hepatol* **43**, 536-543 (2005).

5. Mareux, E. Lapalus, M. Amzal, R. Almes, M. Aït-Slimane, T. Delaunay, J. Adnot, P. Collado-Hilly, M. Davit-Spraul, A. Falguières, T. Callebaut, I. Gonzales, E. Jacquemin, E. Functional rescue of an ABCB11 mutant by ivacaftor: A new targeted pharmacotherapy approach in bile salt export pump deficiency. *Liver Int* **40**, 1917-1925 (2020).

6. Ho, R. H. Leake, B. F. Kilkenny, D. M. Meyer Zu Schwabedissen, H. E. Glaeser, H. Kroetz, D. L. Kim, R. B. Polymorphic variants in the human bile salt export pump (BSEP; ABCB11): functional characterization and interindividual variability. *Pharmacogenet Genomics* **20**, 45-57 (2010).

7. Kubitz, R., Dröge, C., Stindt, J., Weissenberger, K. & Häussinger, D. The bile salt export pump (BSEP) in health and disease. *Clin Res Hepatol Gastroenterol* **36**, 536-553 (2012).

8. Strautnieks, S. S. *et al.* Severe bile salt export pump deficiency: 82 different ABCB11 mutations in 109 families. *Gastroenterology* **134**, 1203-1214 (2008).

9. Dröge, C. *et al.* Sequencing of FIC1, BSEP and MDR3 in a large cohort of patients with cholestasis revealed a high number of different genetic variants. *J Hepatol* **67**, 1253-1264 (2017).
